# Prediction Model for Sensory Perception Abnormality in Autism Spectrum Disorder

**DOI:** 10.3390/ijms24032367

**Published:** 2023-01-25

**Authors:** Zhe Ma, Lisha Xu, Qi Li, Xiang Li, Yaxin Shi, Xirui Zhang, Yuan Yang, Jia Wang, Lili Fan, Lijie Wu

**Affiliations:** Department of Children’s and Adolescent Health, Public Health College, Harbin Medical University, Harbin 150081, China

**Keywords:** autism spectrum disorder (ASD), bioinformatical analysis, sensory perception, differentially expressed genes (DEGs), prediction model

## Abstract

Autism spectrum disorder (ASD) is a neurodevelopmental disorder characterized by heterogeneous clinical phenotypes. Patients often experience abnormal sensory perception, which may further affect the ASD core phenotype, significantly and adversely affecting their quality of life. However, biomarkers for the diagnosis of ASD sensory perception abnormality are currently elusive. We sought to identify potential biomarkers related to ASD sensory perception abnormality to construct a prediction model that could facilitate the early identification of and screening for ASD. Differentially expressed genes in ASD were obtained from the Gene Expression Omnibus database and were screened for genes related to sensory perception abnormality. After enrichment analysis, the random forest method was used to identify disease-characteristic genes. A prediction model was constructed with an artificial neural network. Finally, the results were validated using data from the dorsal root ganglion, cerebral cortex, and striatum of the BTBR T+ Itpr3tf/J (BTBR) ASD mouse model. A total of 1869 differentially expressed genes in ASD were screened, among which 16 genes related to sensory perception abnormality were identified. According to enrichment analysis, these 16 genes were mainly related to actin, cholesterol metabolism, and tight junctions. Using random forest, 15 disease-characteristic genes were screened for model construction. The area under the curve of the training set validation result was 0.999, and for the model function validation, the result was 0.711, indicating high accuracy. The validation of BTBR mice confirmed the reliability of using these disease-characteristic genes for prediction of ASD. In conclusion, we developed a highly accurate model for predicting ASD sensory perception abnormality from 15 disease-characteristic genes. This model provides a new method for the early identification and diagnosis of ASD sensory perception abnormality.

## 1. Introduction

Autism spectrum disorder (ASD) is a neurodevelopmental disorder characterized by social and communication deficits, as well as stereotypical behaviors. The incidence is increasing annually. A recent study has shown that the prevalence of ASD in 4-year-old children is 1.7%, which is higher than the 1.56% reported in the past 2 years [1]. In addition to the aforementioned core symptoms, ASD is often accompanied by sensory perception abnormality, possibly involving vision, smell, hearing, taste, touch, and pain, which are present in 95% of patients with ASD [2]. Patients with ASD could be severely affected by sensory perception abnormality. For example, hearing impairment may aggravate the core symptom phenotype in ASD patients [3] and lead to higher-level communication difficulties [4]; ASD patients also exhibit picky eating behaviors, which can lead to an increased risk of weight gain, obesity, and chronic diseases [5]. Moreover, ASD patients who are insensitive to pain exhibit self-harming behavior [6]. Therefore, sensory perception abnormality in ASD has attracted wide attention and is worthy of further study.

Delayed sensory development in ASD patients can lead to changes in behavioral responses and neural activity patterns and may produce a series of impacts on advanced functions, such as cognition and social interaction [7]. Therefore, the early identification of ASD sensory perception abnormality may be conducive to predicting the risk of ASD in children. The clinical diagnosis of ASD is currently mainly based on medical history, clinical observations, and psychological assessments, such as the Autism Diagnostic Interview-Revised and Autism Diagnostic Observation Schedule [8,9]. However, these criteria are limited, diagnostic methods lack objectivity, and ASD is frequently misdiagnosed. As several children with ASD are diagnosed late [10], after the period of highest brain plasticity, possible interventions are less effective. Therefore, the early identification and diagnosis of ASD sensory perception abnormality are critical.

The etiology of ASD is unknown at present, and research has focused on genetics. The pathogenesis of ASD is complicated, and variants in hundreds of genes, such as *SH2B1*, *KDM5A*, and *DYRK1A*, have been confirmed to be highly correlated with this disorder [11,12,13]. Genes associated with sensory perception in ASD have also been studied. For example, *CNTN6* mutations are a risk factor for ASD auditory and sensory issues [14], and *CNTNAP2* is associated with ASD sensory perception abnormality [15]. However, the major genes or gene combinations responsible for ASD and ASD sensory perception abnormality have not yet been identified up to now.

Bioinformatics techniques are increasingly used to identify hub genes closely related to the presence and development of various diseases [16,17,18]. For example, the use of different microarray platforms to detect gene expression levels in tissues or blood is an emerging method for studying disease mechanisms or diagnosing diseases. Many studies have been conducted using bioinformatics in the field of ASD. Through bioinformatics analysis of the brain tissue and blood data of the ASD population available in databases, many studies have obtained differentially expressed genes (DEGs) and their biological functions [19] and explored the genes and mechanisms related to the molecular pathology of ASD [20]. In this way, it has been found that *EGFR*, *ACTB*, *RHOA*, etc. may be hub genes in ASD [21]. However, to the best of our knowledge, there has been no bioinformatics analysis in the field of sensory perception in ASD. In addition, due to the heterogeneity of clinical phenotypes, it remains unclear which genes are the major genes involved in ASD. ASD should be studied according to the classification of clinical phenotypes, in particular for the early identification of the subtype of sensory perception abnormality, which is an urgent problem.

**Figure 1:**
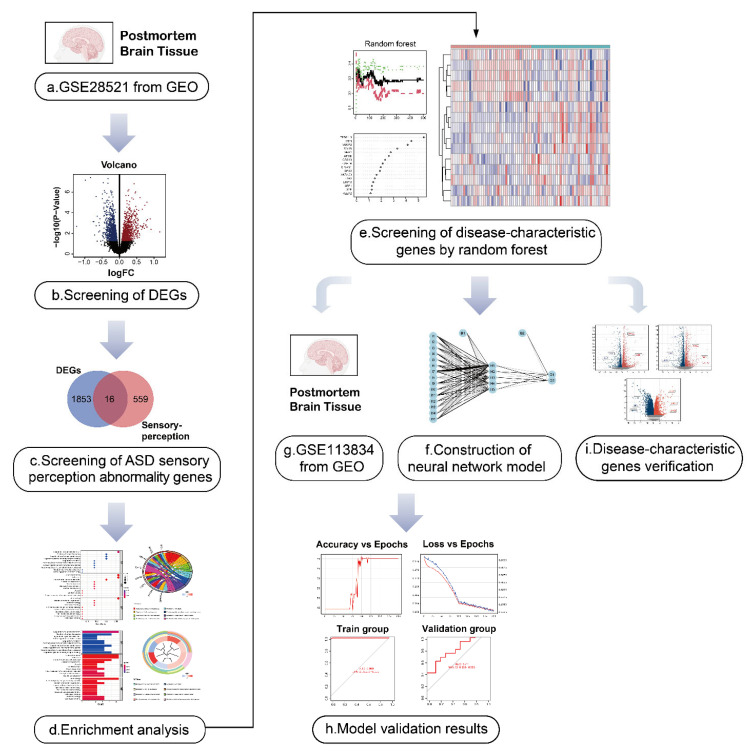
Flow diagram of the study. (**a**,**b**) The autism spectrum disorder (ASD)-related dataset GSE28521 from human post-mortem brain tissues containing 79 samples (40 Controls/39 ASD) was divided into an ASD group and the control group, and the differentially expressed genes (DEGs) in ASD were obtained by differential analysis. The volcano plot shows the up-regulated and down-regulated DEGs. I We took the intersection of ASD DEGs and the sensory perception gene set, and identified the genes associated with sensory perception abnormality in ASD. (**d**) Gene ontology (GO) and Kyoto Encyclopedia of Genes and Genomes (KEGG) enrichment analysis was performed for ASD sensory perception abnormality-related genes to explore their biological function.(**e**) The first 15 ASD sensory perception abnormality-related genes were selected as disease-characteristic genes by random forest analysis to construct the neural network model. (**f**–**h**) Neural network construction: the GSE28521 dataset was used as the training set of the neural network model, and another dataset GSE113834 (27 samples from post-mortem brain tissues, 12 Controls/15 ASD) was used as the validation set. Finally, the area under the curve (AUC) of the model validation result was 0.711. (**i**) The data of the dorsal root ganglion (DRG), cerebral cortex and striatum from BTBR mice were used for the identification of DEGs. Nine genes (*MSN*, *APOE*, *CIB2*, *LRP10*, *LRP1*, *SYP, MPP1, MYH9, and GPC2)* were found among the DEGs, which were consistent with the disease-characteristic genes, verifying the accuracy of the model genes.

Herein, we used data from the Gene Expression Omnibus (GEO) database to screen for genes involved in ASD sensory perception and integrated these genes to establish the first artificial neural network model of ASD sensory perception abnormality. In addition, we used the recognized mouse model of ASD, the BTBR T+ Itpr3tf/J (BTBR) mouse, for verification of our findings. The analytical processes used in our study are shown in Figure 1. The results of this study may be useful for the early identification and diagnosis of sensory perception abnormality in ASD, and it may provide some insights into early targeted intervention in children with ASD.

## 2. Results

### 2.1. Identification of the Sensory Perception Abnormality-Related Genes in ASD

**Figure 2:**
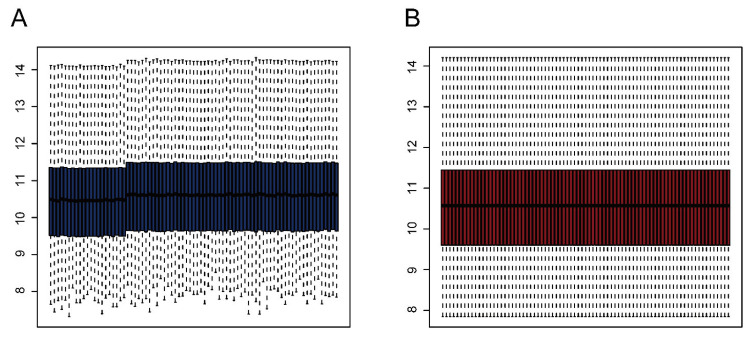
Data correction. (**A**) Box plot before data correction. (**B**) Box plot after data correction.

**Figure 3:**
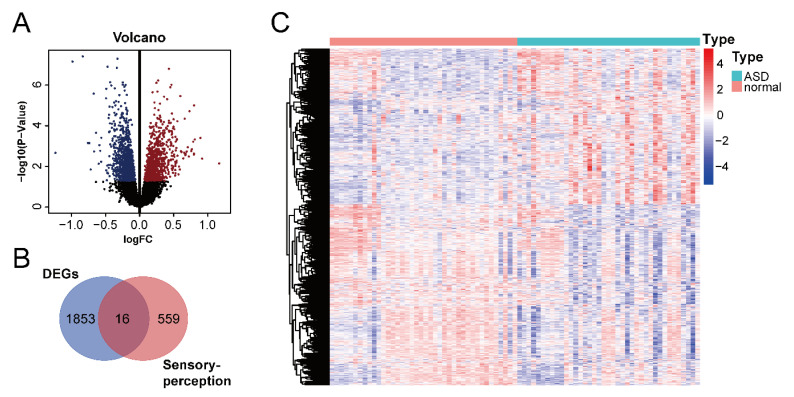
Identification of DEGs in ASD and ASD sensory perception abnormality-related genes. (**A**) Volcano plot of DEGs in ASD. The red color represents up-regulated genes, and the blue color represents down-regulated genes. (**B**) Venn diagram of DEGs in ASD and sensory perception genes. (**C**) Heatmap of DEGs in ASD. Red indicates the upregulated genes, and blue indicates the downregulated genes.

We downloaded the GSE28521 dataset from the GEO database, which was derived from a transcriptome analysis of the autistic brain [20], to search for DEGs in ASD. The GSE28521 dataset is based on a large sample size of human post-mortem brain tissues (Controls: n = 40; ASD: n = 39). Moreover, this dataset has been used to study cognitive impairments in ASD previously [22]. After pre-processing, data correction was performed on the GSE28521 dataset. Figure 2A and B show the box plots before and after data correction, respectively. A total of 1869 DEGs in ASD were identified from the GSE28521 dataset (Figure 3A), and a heatmap was drawn (Figure 3C).

We downloaded the sensory perception gene set from the Molecular Signatures Database v7.5.1 to identify sensory perception abnormality-related genes in ASD. A cross-analysis of ASD DEGs and the sensory perception gene set by means of a Venn analysis resulted in the identification of 16 ASD sensory perception abnormality-related genes (Figure 3B).

### 2.2. Enrichment Analysis

**Figure 4:**
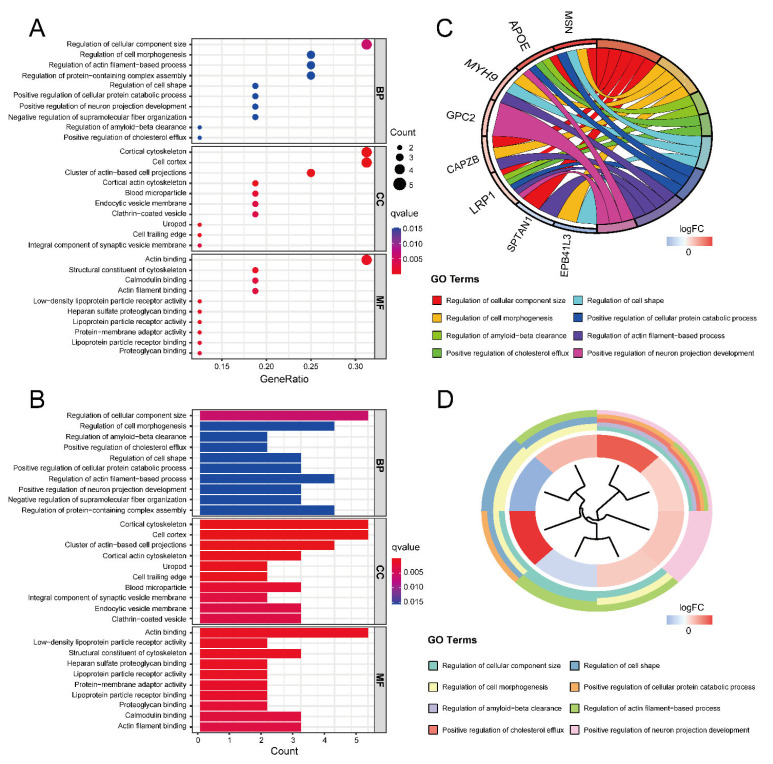
The results of GO enrichment analysis. (**A**) GO enrichment bubble plot, which includes a biological process (BP), molecular function (MF) and cellular component (CC) categories. The *x*-axis represents the gene ratio, and the *y*-axis represents the q-value of different GO items. The size of the bubble indicates the number of genes enriched for each GO term. The redder the color, the smaller the q-value. (**B**) GO enrichment bar graph. The redder the color, the smaller the q-value. (**C**) GO enrichment chord plot. Genes are shown on the left, where red gene bands indicate up-regulated expression, and blue gene bands indicate down-regulated expression. The different colored bars on the right represent different GO terms. Lines connect genes and their corresponding GO terms. (**D**) GO enrichment phylogenetic plot. The bands with different colors in the innermost circle represent different genes, and the bands with different colors in the outer circle represent the GO items enriched by the corresponding genes.

**Figure 5:**
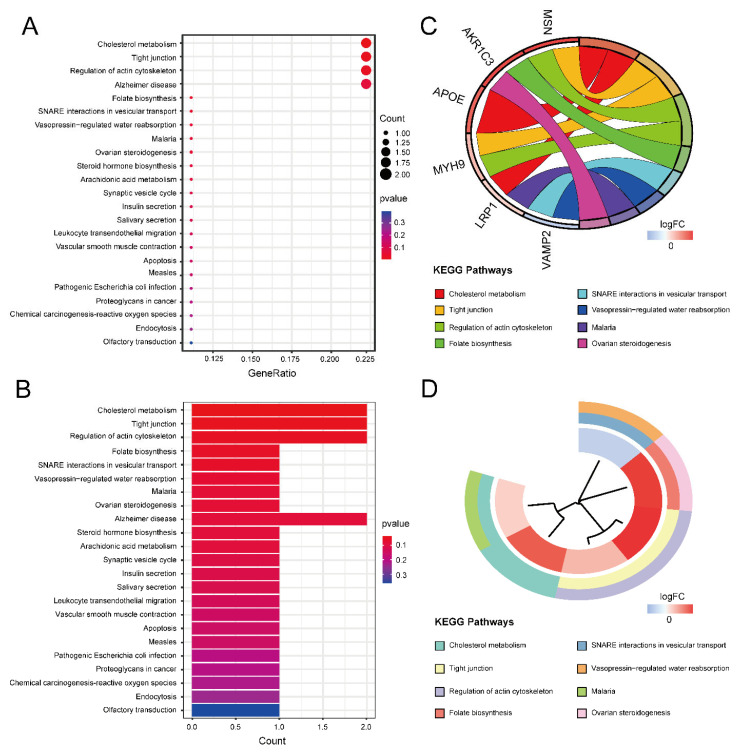
The results of KEGG enrichment analysis. (**A**) KEGG enrichment bubble plot. The *x*-axis represents the gene ratio, and the *y*-axis represents the *p*-value of different pathways. The size of the bubble indicates the number of genes enriched in each KEGG pathway. The redder the color, the smaller the *p*-value. (**B**) KEGG enrichment bar graph. The redder color indicates a smaller *p*-value. (**C**) KEGG enrichment chord plot. Genes are shown on the left, where red gene bands indicate up-regulated expression, and blue gene bands indicate down-regulated expression. The different colored bands on the right represent different pathways. Lines connect genes and their corresponding pathways. (**D**) KEGG enrichment phylogenetic plot. The bands with different colors in the innermost circle represent different genes, and the bands with different colors in the outer circle represent the enriched pathways of the corresponding genes.

Gene Ontology (GO) and Kyoto Encyclopedia of Genes and Genomes (KEGG) enrichment analyses were performed to obtain the function of the 16 ASD sensory perception abnormality-related genes. Figure 4A,B show the enrichment results in the three GO categories. We found enriched genes mainly in the cortical cytoskeleton and cell cortex in the cellular component (CC) terms and actin binding in the molecular function (MF) terms. Figure 4C,D show some GO-enriched terms and significant genes; the genes were mainly enriched in the regulation of cellular component size, regulation of cell morphogenesis, and regulation of actin filament-based processes. Figure 5 shows the KEGG results. The enrichment primarily involved cholesterol metabolism, tight junctions, and regulation of the actin cytoskeleton.

### 2.3. Random Forest Screening for Disease-Characteristic Genes

Using the random forest classifier, we accurately identified disease-characteristic genes among the 16 ASD sensory perception abnormality-related genes. The best number of variables for the binary tree and the average error rate were calculated by performing cyclic random forest classification. Finally, 15 was selected as the ideal number of variables.

**Figure 6:**
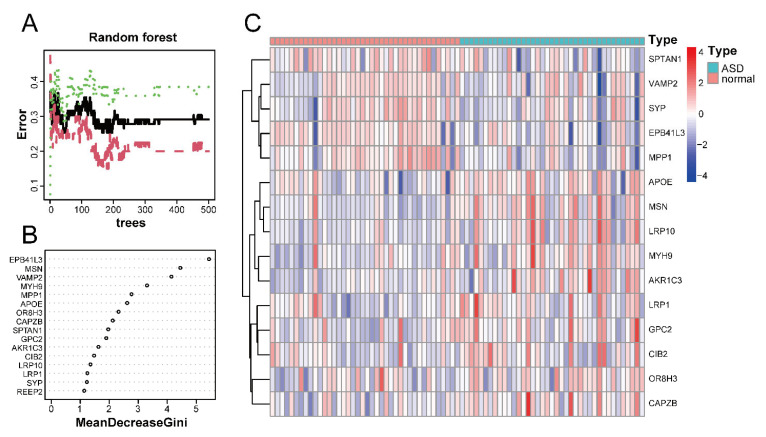
Screening of disease-characteristic genes by using a random forest classifier. (**A**) The relationship between the number of decision trees and the error rate. The *x*-axis and *y*-axis represent the number of decision trees and the error rate, respectively. The green color represents the error in the control group, the red color represents the error in the experimental group, and the black color represents the error in all samples. The error rate is lowest when the number of decision trees is 43. (**B**) Feature importance was measured using the Gini coefficient method. The *x*- and *y*-axes represent the genetic variable and the importance index, respectively. (**C**) Heatmap of 15 disease-characteristic genes obtained by random forest analysis. Red indicates the upregulated genes, and blue indicates the downregulated genes.

Figure 6A shows the relationship between the number of decision trees and the error rate. As shown in Figure 6B, *EPB41L3* and *MSN* were identified as the most relevant genes, followed by *VAMP2*, *MYH9*, *MPP1*, and *APOE*. Based on the top 15 candidate genes, we performed unsupervised clustering on the GSE28521 dataset and drew a heatmap (Figure 6C), which showed that these 15 genes could distinguish between ASD and control samples among the 79 samples in the GSE28521 dataset.

### 2.4. Construction of the ASD Sensory Perception Abnormality Prediction Model

**Figure 7:**
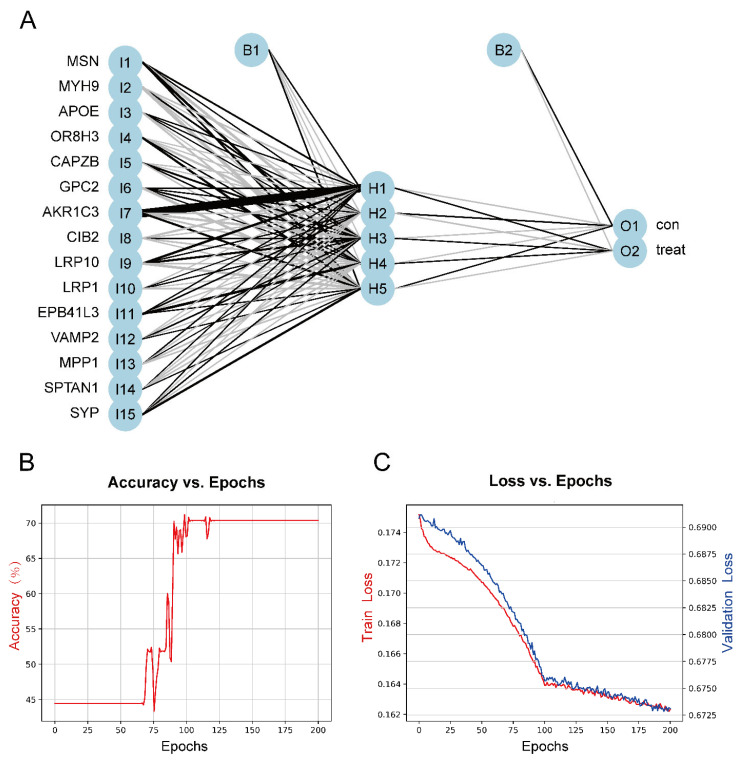
(**A**) The visualization of the neural network results. (**B**) Figure of accuracy vs. epochs. (**C**) Figure of loss vs. epochs.

To construct a model for predicting sensory perception abnormality in ASD, data were normalized using the Z-score, and then the number of hidden layers was set to 5. The results of the model were evaluated by dividing the datasets into training and validation datasets. Candidate gene indicator weights were determined using the training set. The classification efficiency of the model scores, which were constructed based on gene expression and weights, was validated using the validation set. Figure 7A shows the visualization of the ASD prediction model results. Figure 7B,C show the accuracy versus epochs and the loss versus epochs of the model, respectively.

**Figure 8:**
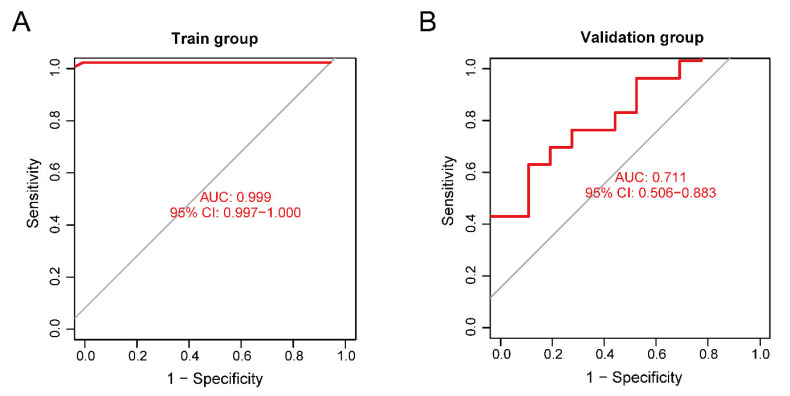
(**A**) Receiver operating characteristic (ROC) curve of the training set. (**B**) ROC curve of the validation set.

The training set validation result was used as the model classification performance, as shown by the receiver operating characteristic (ROC) curve in Figure 8A. The area under the ROC curve (AUC) was close to 1 (0.999), indicating high model accuracy.

We selected another GEO dataset, GSE113834, to verify the accuracy of our model as a validation set for functional verification. We calculated the maximum and minimum values after normalized data processing, evaluated the classification efficiency, and compared the AUCs. The validation result of the validation set was considered as the model classification performance, as shown by the ROC curve in Figure 8B. The AUC was 0.711, confirming that the model prediction accuracy was high.

For the performance of our neural network model, the ROC of the validation set did not show over-fitting, but the accuracy of the model was limited by the small sample size of the validation set.

### 2.5. Disease-Characteristic Genes Verification

**Figure 9:**
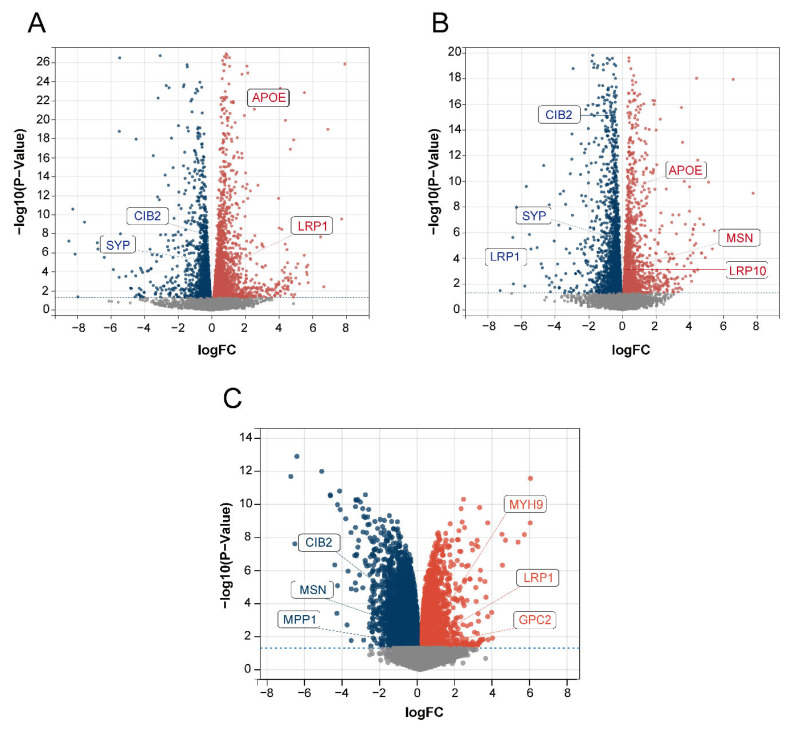
Volcano plot of DEGs. The red color represents up-regulated genes, and the blue color represents down-regulated genes. (**A**) Data 1 DEGs volcano plot. (**B**) Data 2 DEGs volcano plot. (**C**) GSE156646 DEGs volcano plot.

We used the dorsal root ganglion (DRG; the tissue most strongly associated with the pain and touch in sensory perception) of BTBR mice and C57BL/6J (B6) control mice for RNA analysis to obtain DEGs to verify the relationship between the 15 identified disease-characteristic genes and the DEGs. Among the 15 genes, *MSN*, *APOE*, *CIB2*, *LRP10*, *LRP1*, and *SYP* were all present among the DEGs in our data (Figure 9A,B). In addition, we used the GSE156646 dataset from the cerebral cortex and striatum (the tissue most strongly associated with sight, hearing, and smell in sensory perception) of BTBR mice and B6 control mice to obtain DEGs to verify the 15 disease-characteristic genes. Among the 15 genes, *CIB2*, *MSN*, *MPP1*, *MYH9*, *LRP1*, and *GPC2* were also present among the DEGs in this data (Figure 9C). The above results further confirmed the reliability of our gene screening and artificial neural network model.

## 3. Discussion

ASD includes a wide range of developmental disorders of the nervous system. Sensory perception abnormality has a severe impact on patients and their families and has received widespread attention. Currently, there are no genes or genomes that can be used as biomarkers for sensory perception abnormality in ASD. In this study, we explored DEGs related to the sensory perception abnormality in ASD, some of which were also validated in BTBR mice. No such genes have been reported previously. The prediction model constructed using these genes showed high accuracy and provided an important basis for the diagnosis of sensory perception abnormality in ASD. These results could also offer new possibilities for the early identification and prediction of sensory perception abnormality in ASD.

ASD is a group of developmental disabilities with heterogeneous clinical manifestations, and it likely encompasses multiple subgroups of disease pathologies and etiologies. In previous studies, researchers did not differentiate between the ASD phenotypes when searching for ASD susceptibility genes. Many genes related to the development and function of neuron synapses, for example, *SCN2A* and *POGZ*, were considered to be susceptibility genes for ASD [23,24,25]. However, our study mainly focused on sensory perception abnormality in ASD. Surprisingly, we identified 16 DEGs of sensory perception abnormality in ASD that were enriched in actin, cholesterol metabolism, and tight junctions, which differed from ASD susceptibility genes screened from the overall ASD phenotypes. These findings further support the recognized heterogeneity of ASD and underscore the necessity of phenotyping research in ASD. We also found that the corresponding enrichment result of the 16 DEGs was strongly correlated with synaptic plasticity and synaptic proteins. Synaptic plasticity is regulated by actin polymerization [26]. Several studies have shown that synaptic loss and subsequent cognitive deficits can be attributed to disruptions of brain cholesterol metabolism in Alzheimer’s disease (AD) pathology [27]. A bioinformatics and proteomics analysis of epithelial tight junctions has revealed that synaptic proteins and signaling molecules are associated with tight junctions [28]. In addition to synaptic function-related genes (*SYP* [29] and *CIB2* [30]), other disease-characteristic genes, such as *LRP1* [31], *SPTAN1* [32], *MSN* [33], *APOE* [34], and *VAMP2* [35], also indirectly regulate synaptic development and function. This is consistent with the finding that most ASD model mice have an abnormal synaptic transmission as well as abnormal sensory perception. It is possible that the mechanism of action of the genes we identified as related to ASD sensory perception abnormality involves synaptic function and development. This also provides a good direction for future research on the underlying mechanism.

Several reports have shown that lipid metabolism has important implications for maintaining the normal physiological function of synaptic neurons [36,37]. Apolipoprotein E (APOE) promotes the clearance of plasma lipoproteins rich in triglycerides and cholesterol [38]. As one of the six ASD and AD risk genes, *APOE* is also involved in regulating ASD biomarkers in adults [39,40]. In addition, *APOE* plays an important role in both neural repair and olfactory system function [41]. Based on the present study, *APOE* may also be involved in sensory perception abnormality in ASD, providing ideas for subsequent research. As a member of the low-density lipoprotein receptor family, LDL receptor-related protein 10 (LRP10) is involved in endocytosis, lipid metabolism, and amyloid-precursor protein transport and processing [42]. LDL receptor-related protein 1 (LRP1) is one of the APOE receptors that is highly expressed in neurons, astrocytes, and so on [43]. *LRP1* is associated with high-functioning ASD and is also a susceptibility gene for schizophrenia [44,45]. *LRP1* knockout in adult mouse neurons results in impaired brain lipid metabolism, progressive age-dependent synapse loss, and neurodegeneration [46]. In this study, we revealed that *LRP10* is closely related to ASD and sensory perception. Taken together, abnormal lipid metabolism may be considered as a direction for future research on ASD sensory perception abnormality, and *APOE*, *LRP10*, and *LRP1* in particular may be useful research targets.

Sensory perception involves sight, hearing, smell, taste, touch, and pain, and it involves both the peripheral and central nervous systems. Sensory signals are transmitted from the peripheral nervous system to the brain [47], where the dorsal root ganglion (DRG) is the first station of sensory transmission. The DRG plays an important role in the processing of pain and touch in sensory perception [48]. We analyzed the RNA data extracted from the DRG of ASD model BTBR mice and identified the DEGs. Among the DEGs, *MSN*, *APOE*, *CIB2*, *LRP10*, *LRP1*, and *SYP* coincided with the 15 disease-characteristic genes we had identified. These genes may be associated with abnormal touch and pain perception in ASD. In addition, the cerebral cortex and striatum play important roles in vision, hearing, and smell [49,50,51,52,53]. We analyzed the dataset from the cerebral cortex and striatum of BTBR ASD model mice and identified the DEGs as compared to B6 mice. We found that, among the DEGs, *CIB2*, *MSN*, *MPP1*, *MYH9*, *LRP1*, and *GPC2* were also present among the 15 disease-characteristic genes. These genes may be associated with abnormal visual, auditory, and olfactory functions in ASD. Validation of the ASD model mice confirmed that the genes we had identified were strongly related to ASD sensory perception.

Currently, the screening and clinical diagnosis of ASD still rely on scales and questionnaires completed after clinical observation or evaluation, which are often subjective and prone to misdiagnosis. With the growing recognition of the importance of early diagnosis to enhance the benefit from treatment, increasing efforts are being made to explore other possible biomarkers for ASD. Several studies have used brain imaging data to implement machine-learning in ASD prediction or diagnosis models, such as ASD-SAENet [54], DarkASDNet [55], and ASD-DiagNet [56]. Other researchers have proposed different diagnostic models for ASD, such as Grossi et al. [57], who established an “MS-ROM/IFAST” model that allows the distinction of children with ASD from those with other neuropsychiatric disorders. Zhang et al. [58] diagnosed ASD by identifying methylation markers. Wang et al. [59] predicted ASD by identifying genetic variations. In other studies, eye-fixation data [60], eye-tracking data from face-to-face conversations [61], and images of children’s faces [62] have been used for ASD diagnosis. The above studies have proposed new concepts and diagnostic methods, but they have some limitations. In particular, as ASD symptoms are heterogeneous, a disease prediction model constructed without distinguishing clinical phenotypes may affect early diagnosis performance. Based on bioinformatics, we used microarray data to detect gene expression levels to construct a model for predicting sensory perception abnormality related to ASD, which has not been reported in previous studies. This model has some practical, potential application value. We built a model based on genes related to sensory perception abnormality through a neural network, which is more specific to sensory perception abnormality in ASD. The screened sensory perception abnormality-related genes are associated with synaptic transmission and lipid metabolism, which may provide a basis for the study of mechanisms underlying ASD sensory perception abnormality. In the future, specific gene expression data may be input into the model to predict changes in the sensory perception of individuals with ASD. This method of prediction based on phenotype classification may improve the accuracy of early identification and screening so that children with ASD can receive timely intervention and improvements to their quality of life.

However, our study had some limitations. It is necessary to increase the number of data samples for verification. We also need to increase the clinical and prognostic information of the sample and use more comprehensive data information to build the model. RNA-seq, whole exome sequencing, single-cell sequencing, and spatial transcriptome sequencing should be performed on the original samples. We plan to explore gene mutations at the DNA level and gene expression in cell populations at the single-cell level from the RNA transcriptome level and study the localization of different genes expressions at the spatial transcriptome level to verify prediction models from a multi-omics perspective. Further experiments are needed to verify the expression and functional mechanisms associated with the identified genes.

## 4. Materials and Methods

### 4.1. Data Download and Processing

Gene expression profiles were downloaded as raw data from the GSE28521, GSE113834, and GSE156646 datasets of the public repository, GEO (https://www.ncbi.nlm.nih.gov/geo/ (accessed on 5 March 2022)). The GEO database collects raw and processed data for high-throughput gene expression and genomic research; contains various data, including gene-chip, transcriptome sequencing, and methylation-chip; and has been widely used for the mining and verification of disease-related data [63].

The GSE28521 dataset (*Homo sapiens*) from GPL6883 comprises 79 samples, including 39 ASD and 40 control samples. The GSE113834 dataset (*Homo sapiens*) from GPL15207 comprises 27 samples, including 15 ASD and 12 control samples. The GSE156646 dataset (*Mus musculus*) from GPL21163 comprises eight samples, including four ASD and four control samples. All available samples were included in the study. The details of the three datasets are presented in Table 1.

### 4.2. Identification of ASD DEGs

DEGs were screened by differential expression analysis of the GSE28521 dataset using R software (version 4.1.2, *Lucent Technologies*, *Murray Hill*, *N.J.*, *USA*, https://www.r-project.org/ (accessed on 5 March 2022)). The samples were divided into ASD and normal groups. The R package limma [64] was used to analyze differential expression. This package employs a linear model approach to analyze the differential expression between groups. DEGs were screened at a *p*-value < 0.05. The results were visualized using the R package pheatmap [65] for the heatmap and ggplot2 [66] for the volcano plot.

### 4.3. Identification of the Sensory Perception Abnormalit-Related Genes in ASD

The Molecular Signatures Database v7.5.1 (https://www.gsea-msigdb.org/gsea/msigdb (accessed on 5 March 2022)) was used to obtain the sensory perception gene set (REACTOME_SENSORY_PERCEPTION). An online tool called Draw Venn Diagram (http://bioinformatics.psb.ugent.be/webtools/Venn/ (accessed on 5 March 2022)) was used to obtain ASD sensory perception abnormality-related genes by combining the sensory perception gene set and ASD DEGs.

### 4.4. Enrichment Analysis

The clusterProfiler package [67] was used to perform GO and KEGG enrichment analyses. GO analysis includes three categories: biological process (BP), molecular function (MF), and cellular component (CC). The GO enrichment cutoff was a q-value of <0.05, and the KEGG enrichment cutoff was a *p*-value of < 0.05.

### 4.5. Random Forest Screening for Disease-Characteristic Genes

Random forest analysis is a popular method for evaluating and classifying features [68,69] that outperforms several typical classifiers such as decision trees and logistic regression models. Random forest classifier model training was implemented using the R package, randomForest (https://cran.r-project.org/package=randomForest (accessed on 5 March 2022)). The point with the lowest error value in the model was selected for gene screening, and the obtained genes were used as disease-characteristic genes for subsequent model development. Finally, we clustered these disease-characteristic genes in the GSE28521 dataset using the R package pheatmap [65] and plotted them as a heatmap.

### 4.6. Construction of the Prediction Model for ASD Sensory Perception Abnormality

The GSE28521 and GSE113834 datasets were used for the training and validation of the neural network models. The R package sva [70] “combat” function was used to remove batch effects and all expression quantities were normalized using the Z-score. We selected the top 15 most important genes in the random forest for the construction of neural networks.

First, we benchmarked the default parameters of several common classification models, among which the neural network model had the best performance; therefore, we chose the neural network model for modeling. The PyTorch framework based on Python (version 3.8.1, *Wilmington*, *D.E.*, *USA*, https://www.python.org/ (accessed on 5 March 2022)) was used for the neural network model. Accuracy and loss were recorded during modeling. The Python package matplotlib [71] was used for visualization to observe the model-fitting efficiency. After optimizing the superparameters, the parameters of the neural network model were set as follows: number of hidden layers = 5, epoch = 400, learning rate = 0.1, and batch size = 8.

The validation results of the classification performance were calculated using the R package pROC [72]. The validity of the prediction model was verified using the validation set GSE113834. Additionally, the ROC curve and AUC were used to verify the classification efficiency.

### 4.7. Verification of Disease-Characteristic Genes

We extracted the DRG from BTBR (a preclinical mouse model of the core autism symptom domains) and B6 (control strain for BTBR) mice for RNA-seq analysis to explore the ASD DEGs and validate our disease-characteristic genes screened using random forest. The details of the two datasets are listed in Table 2. In addition, the GEO dataset GSE156646 was also used to verify disease-characteristic genes, which were derived from the cerebral cortex and striatum of BTBR and B6 mice. The details of these data are shown in Table 1. The above results were visualized in volcano plots obtained using the R package ggplot2 [66]. DEGs were screened at a *p*-value < 0.05.

## 5. Conclusions

In this study, we identified 16 DEGs related to sensory perception abnormality in ASD. Enrichment analysis showed that these genes were mainly enriched in actin, cholesterol metabolism, and tight junction. This suggested that the mechanism of sensory perception abnormality in ASD may be related to synaptic development and function. We used random forest analysis to identify 15 disease-characteristic genes, nine of which (*MSN*, *APOE*, *CIB2*, *LRP10*, *LRP1*, *SYP*, *MPP1*, *MYH9*, and *GPC2*) were verified in animal models, which further proved the reliability of the identified genes and the prediction model. The use of a bioinformatic method to construct an accurate sensory perception prediction model in ASD for predicting a single clinical phenotype of ASD has not been reported previously. Our results provide important directions for research in the field of sensory perception abnormality in ASD, and they also provide new ideas for the early identification and screening of sensory perception abnormality in ASD.

## Figures and Tables

**Table 1:** The characteristics of the three datasets in the study.

GEO Datasets	Country	Experiment Type	Organization Type	Sample Size (Controls/ASD)	Platform
GSE28521	USA	Expression profiling by array	*Homo sapiens*; Postmortem brain tissue: Cerebellum, Frontal cortex, and Temporal cortex	79 (40/39)	GPL6883
GSE113834	Spain	Expression profiling by array	*Homo sapiens*; Postmortem prefrontal cortex tissue	27 (12/15)	GPL15207
GSE156646	Japan	Expression profiling by array	*Mus musculus*; Cerebral cortex and Striatum	8 (4/4)	GPL21163

**Table 2:** The characteristics of the RNA-seq data in the study.

Data	Experiment Type	Organization Type	Sample Size (B6/BTBR)
Data 1	Expression profiling by high throughput sequencing	*Mus musculus* (6 weeks old, male); DRG	6 (3/3)
Data 2			8 (4/4)

## Data Availability

Publicly available datasets were analyzed in this study. This data can be found here: [http://www.ncbi.nlm.nih.gov/geo (accessed on 5 March 2022)].
